# A flexible MRI coil based on a cable conductor and applied to knee imaging

**DOI:** 10.1038/s41598-022-19282-6

**Published:** 2022-09-02

**Authors:** Bili Wang, Syed S. Siddiq, Jerzy Walczyk, Mary Bruno, Iman Khodarahmi, Inge M. Brinkmann, Robert Rehner, Karthik Lakshmanan, Jan Fritz, Ryan Brown

**Affiliations:** 1 Department of Radiology, Center for Advanced Imaging Innovation and Research (CAI2R), New York University Grossman School of Medicine, 660 First Ave, New York, NY USA; 2 Medical Physics, Memorial Sloan Kettering Cancer Center, New York, NY USA; 3 Division of Musculoskeletal Radiology, Department of Radiology, New York University Grossman School of Medicine, New York, NY USA; 4 Siemens Medical Solutions USA Inc., Malvern, PA USA; 5 Siemens Healthcare GmbH, Erlangen, Germany

**Keywords:** Electrical and electronic engineering, Preclinical research

## Abstract

Flexible radiofrequency coils for magnetic resonance imaging (MRI) have garnered attention in research and industrial communities because they provide improved accessibility and performance and can accommodate a range of anatomic postures. Most recent flexible coil developments involve customized conductors or substrate materials and/or target applications at 3 T or above. In contrast, we set out to design a flexible coil based on an off-the-shelf conductor that is suitable for operation at 0.55 T (23.55 MHz). Signal-to-noise ratio (SNR) degradation can occur in such an environment because the resistance of the coil conductor can be significant with respect to the sample. We found that resonating a commercially available RG-223 coaxial cable shield with a lumped capacitor while the inner conductor remained electrically floating gave rise to a highly effective “cable coil.” A 10-cm diameter cable coil was flexible enough to wrap around the knee, an application that can benefit from flexible coils, and had similar conductor loss and SNR as a standard-of-reference rigid copper coil. A two-channel cable coil array also provided good SNR robustness against geometric variability, outperforming a two-channel coaxial coil array by 26 and 16% when the elements were overlapped by 20–40% or gapped by 30–50%, respectively. A 6-channel cable coil array was constructed for 0.55 T knee imaging. Incidental cartilage and bone pathologies were clearly delineated in T1- and T2-weighted turbo spin echo images acquired in 3–4 min with the proposed coil, suggesting that clinical quality knee imaging is feasible in an acceptable examination timeframe. Correcting for T1, the SNR measured with the cable coil was approximately threefold lower than that measured with a 1.5 T state-of-the-art 18-channel coil, which is expected given the threefold difference in main magnetic field strength. This result suggests that the 0.55 T cable coil conductor loss does not deleteriously impact SNR, which might be anticipated at low field.

## Introduction

Radiofrequency (RF) coils play a key role in the underlying signal-to-noise ratio (SNR) in magnetic resonance imaging (MRI). Traditional coils are built using rigid or semi-rigid conductors based on copper clad circuit board materials, which are enclosed in fixed or semi-flexible housing structures. Copper is the preferred conductor material because its high electrical conductivity preserves the relatively low MRI signal from noise that arises from resistive loss in the coil. Meanwhile, the legacy of fixed housing structures can be traced to the desire to maximize mechanical robustness and provide a predictable geometric framework for coil decoupling^1^. However, the performance of such devices can be compromised by anatomic variation, which can result in lack of comfort, accessibility, and suboptimal SNR^2^. Furthermore, rigid coils are generally unable to accommodate a span of anatomic postures, for example to study musculoskeletal kinetics^3–7^.

To overcome these limitations, a variety of specialized flexible conductors have been recently developed in attempt to replace rigid conductors, many of which are reviewed in Darnell et al.^8^. Among the intriguing options, Corea et al. demonstrated screen-printed coils on a flexible substrate^9,10^ and Jia et al. utilized flexible ribbon cable to form a resonant antenna^11^. While encouraging, both approaches use flat conductors that can make multi-directional flexion difficult. Other methods that offer greater flexibility and even the ability to stretch include braided copper^3^ and silver-coated thread^12^. Stretchable braided copper coils can be difficult to construct because of the need for integration with a substrate such as elastic that precisely restores unmalleable conductor to a neutral contour after deformation. Silver-coated thread is attractive but involves a tradeoff between mechanical flexibility and effective conductivity. Alternatively, Port et al. showed that eutectic gallium indium "liquid metal" or conductive elastomer^4,5^ coils provide excellent electro-mechanical properties and are relatively straightforward to assemble. While these methods appear to represent a major step toward truly wearable coils, the materials remain expensive or difficult to source. Yet another option is to resonate commercially available coaxial cable by carefully selecting its characteristics (i.e. cross-sectional diameter, length, insulating dielectric, and manually-inserted shield openings) in accordance with the operating frequency^6,13–15^. Such designs have been referred to as shielded loop resonators, shielded coaxial cable coils, or high-impedance coils. In this work, we will refer to such coils as “coaxial coils” to distinguish them from “cable coils” that will be described later. While the coaxial cables upon which coaxial coils are based are not elastic, they represent a flexible, ready-made, and inexpensive subgroup of braided copper conductors. Coaxial coils also perform well in fluid mechanical regimes in which the geometry is not fixed^6,15^.

The purpose of this work was to translate coaxial coils to 0.55 Tesla, an environment in which coil conductor losses can be significant. In doing so, we discovered that simply resonating the coaxial cable shield with a capacitor while the inner conductor remained electrically floating created a highly effective coil. To demonstrate the performance of “cable coils”, we measured tuning, decoupling, and SNR as a function of geometry and overlap. We then applied the cable coil concept to a six-channel array for 0.55 T knee MRI, an application that can benefit from flexibility due to the variable nature of the anatomy.

## Results

**Figure 1:**
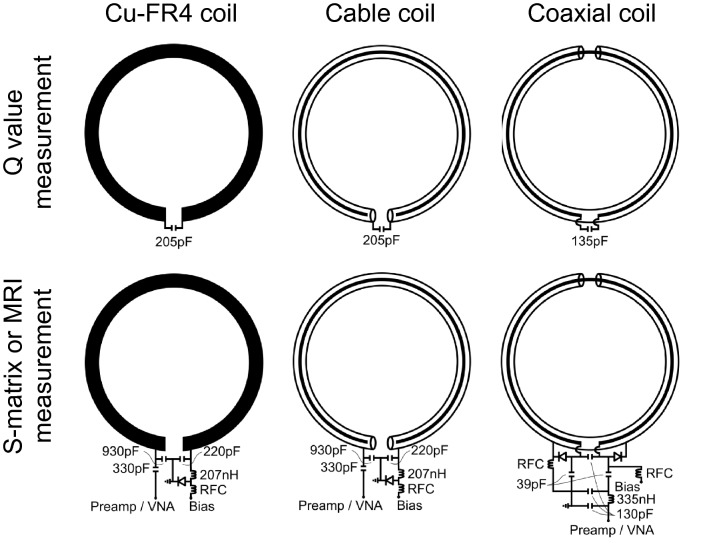
Coil schematics for Q measurements (top row) and S-matrix or MRI measurements (bottom row). A fuse was installed (not shown) in loops used for in vivo experiments. VNA: vector network analyzer. RFC: radiofrequency choke.

Coil loss and loading were investigated by measuring unloaded-to-loaded Q ratios for 10-cm diameter loops tuned to 23.55 MHz (Fig. 1, top row). The Q-ratio of a cable loop built from RG-223 coaxial cable with a floating center conductor was 2.7 compared to 3.0 for a reference loop based on copper clad FR4 circuit board (Cu-FR4) (Table 1). The Q ratios indicate that the relative efficiency of the cable loop was within 3% of the Cu-FR4 loop as defined by $\sqrt {1 - {\text{Q ratio}}^{ - 1} }$^5,16,17^. The relative efficiency of a RG-223 coaxial loop was 26% lower.

**Table 1:** Quality factor (Q), efficiency, and relative efficiency values measured at 23.55 MHz for 10-cm diameter loops built from various conductors.

Coil	Cross-sectional diameter (mm)	Unloaded Q	Loaded Q	Q ratio	Efficiency	Relative efficiency
Cu-FR4	11.0*	325	108	3.0	0.82	1.00
Cable	3.5	310	114	2.7	0.80	0.97
Cable**	3.5	292	114	2.6	0.78	0.96
Coaxial	3.5	126	80	1.6	0.60	0.74

*Width. **Inner conductor and insulation removed.

The loaded Q values were measured while the loops were wrapped onto a 13.5-cm diameter cylindrical dielectric phantom. Efficiency is defined as $\sqrt{1-{\text{Q ratio}}^{-1}}$, while relative efficiency is defined as $\sqrt{1-{\text{Q ratio}}^{-1}}$ normalized to that of the Cu-FR4 loop.

We manually removed the floating center conductor and dielectric insulation to investigate their impact on the cable loop. We found the relative efficiency with or without the center conductor changed by 1%, suggesting that negligible current was present on the inner conductor. While the center conductor and insulation can be easily removed by hand, the cables were kept intact in all other experiments.

**Figure 2:**
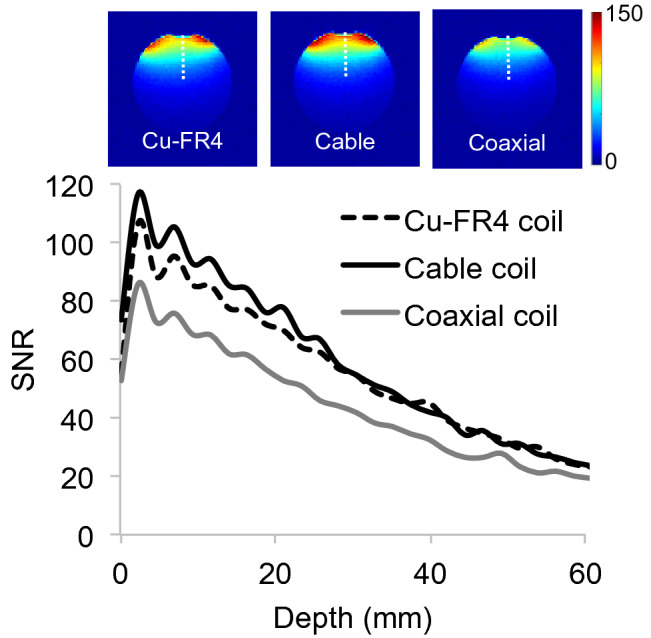
SNR maps (top) and profiles as a function of depth (bottom) in a phantom for Cu-FR4, cable, and coaxial coils. White lines in the maps indicate the position of the profiles.

Interface circuits were installed on the loops in order to perform MRI measurements (Fig. 1, bottom row). The measurements showed that for depths up to 60 mm, the cable and Cu-FR4 loops had similar SNR to within 5% (median SNR difference between pixels at identical depths) along a profile through the loops’ main axes (Fig. 2). The coaxial loop SNR was 34% lower than the cable loop.

**Figure 3:**
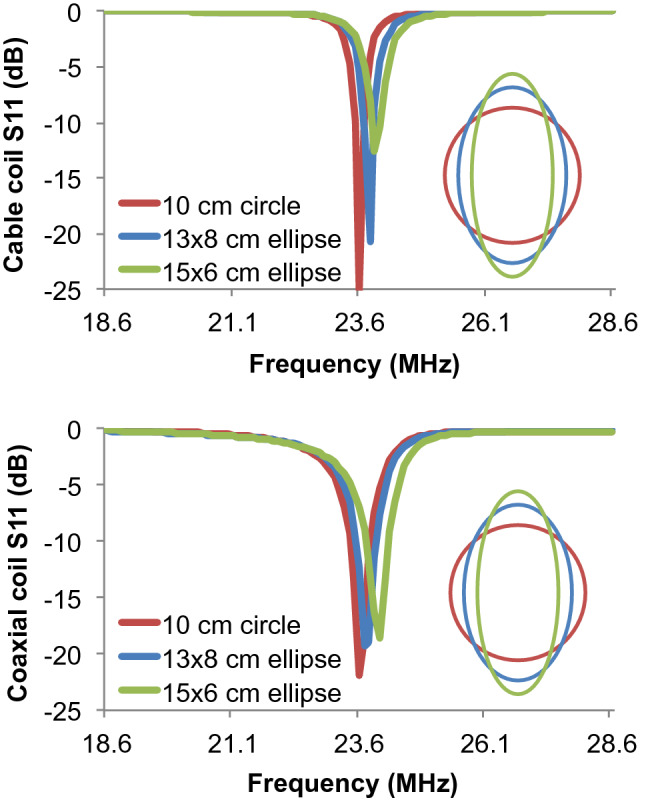
Reflection coefficient (S_11_) as a function of geometry for a cable coil (top) and coaxial coil (bottom). The loops were tuned with circular geometry and wrapped onto a 13.5-cm cylindrical dielectric phantom. The loops were then elongated into ellipses with major (minor) axes of 13-cm (8-cm) and 15-cm (6-cm) without retuning. Scaled representations of the coil contours are overlaid.

**Figure 4:**
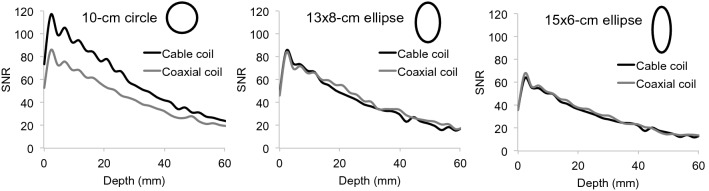
SNR profiles as a function of depth in a phantom for cable and coaxial coils arranged with circular (left), 13 × 8-cm elliptical (middle), and 15 × 6-cm elliptical geometry (right). Scaled representations of the coil contours are overlaid.

We followed the approach laid out in Ruytenberg et al.^15^ to investigate tuning variability with respect to coil geometry. The reflection coefficients of cable and coaxial loops with different levels of elongation are shown in Fig. 3. The cable loop reflection coefficient at 23.55 MHz was − 25.2, − 5.6, and − 3.2 dB when arranged as a circle, 13 × 8-cm ellipse, and 15 × 6-cm ellipse, respectively. For comparison, the reflection coefficient of a coaxial loop, a configuration that has shown robustness against geometric distortion^15^, was − 21.9, − 12.3, and − 7.0 dB for the three geometries. SNR profiles as a function of coil geometry show that the cable and coaxial loops performed similarly when elongated into 13 × 8-cm or 15 × 6-cm ellipses (Fig. 4).

**Figure 5:**
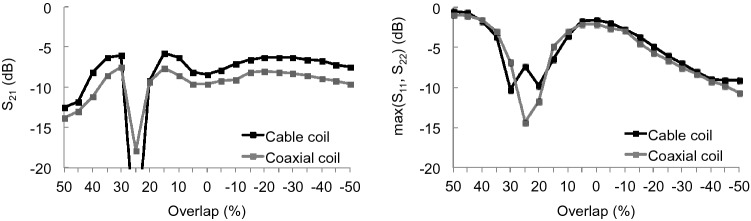
S-matrix measurements as a function of overlap for two-channel arrays based on cable and coaxial loops. Left: transmission coefficient (S_21_). Right: maximum reflection coefficient (S_11_ or S_22_).

The S-matrices for two-channel arrays based on cable or coaxial loops as a function of overlap are shown in Fig. 5. The coaxial loops maintained a lower transmission coefficient than the cable loops over most of the overlap range tested (− 50 to 50 mm). Both configurations had similar worst-case reflection coefficients, which were defined as the maximum of S_11_ and S_22_ for a given overlap.

**Figure 6:**
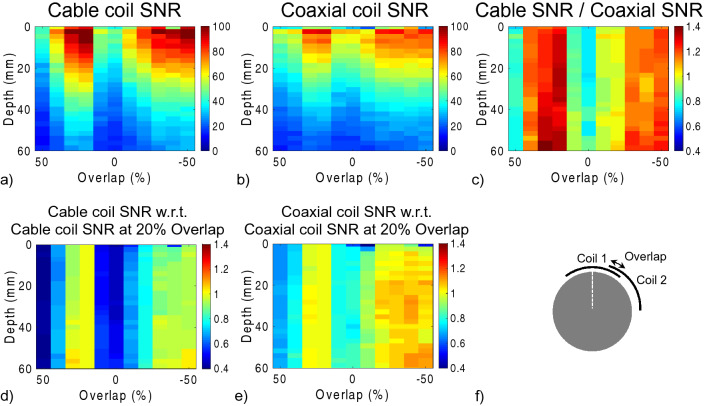
SNR as a function of depth and overlap along the main axis of one coil in two-channel arrays based on cable coils (**a**) or coaxial coils (**b**). Cable coil array SNR normalized by coaxial coil array SNR (**c**). Cable coil SNR normalized by cable coil SNR with 20% overlap (**d**), which is the approximate position that minimized the transmission coefficient (see Fig. 5). Coaxial coil SNR normalized by coaxial coil SNR with 20% overlap (**e**). Overlap is expressed as a percentage of the coil diameter (10-cm). Negative overlaps indicate gaps. Schematic of the setup illustrates the 20% overlap case and shows the SNR profile along the main axis of coil 1 (**f**). Each column of the ratio maps in (**c**–**e**) was smoothed to improve visualization using a median filter with 11-mm kernel.

SNR profiles as a function of depth and coil overlap for two-channel arrays based on cable or coaxial loops are shown in Fig. 6a, b. The arrays were arranged with + 50 to − 50% overlap as in Fig. 6f. The cable coil array was sensitive to overlap; when the overlap was − 10, 0, 10, 40, or 50% the cable coil array SNR dropped by more than 30% from that obtained with the optimal overlap for decoupling (Fig. 6d). In comparison, the cable coil array SNR dropped by more than 30% from that obtained with optimal overlap only when the overlap was set to 50% (Fig. 6e). However, the cable-to-coaxial array SNR ratio in Fig. 6c shows that the cable array outperformed the coaxial array by 26% (median improvement in voxels 0 to 60 mm deep) when the overlap was between 20 and 40%, and by 16% when the overlap was between − 50 and − 30%. The coaxial array outperformed the cable array by 16 and 18% when the overlap was 0 to 10% and 50%, respectively. The SNR for both arrays was the same to within 3% when the overlap was − 20 to -10%.

**Figure 7:**
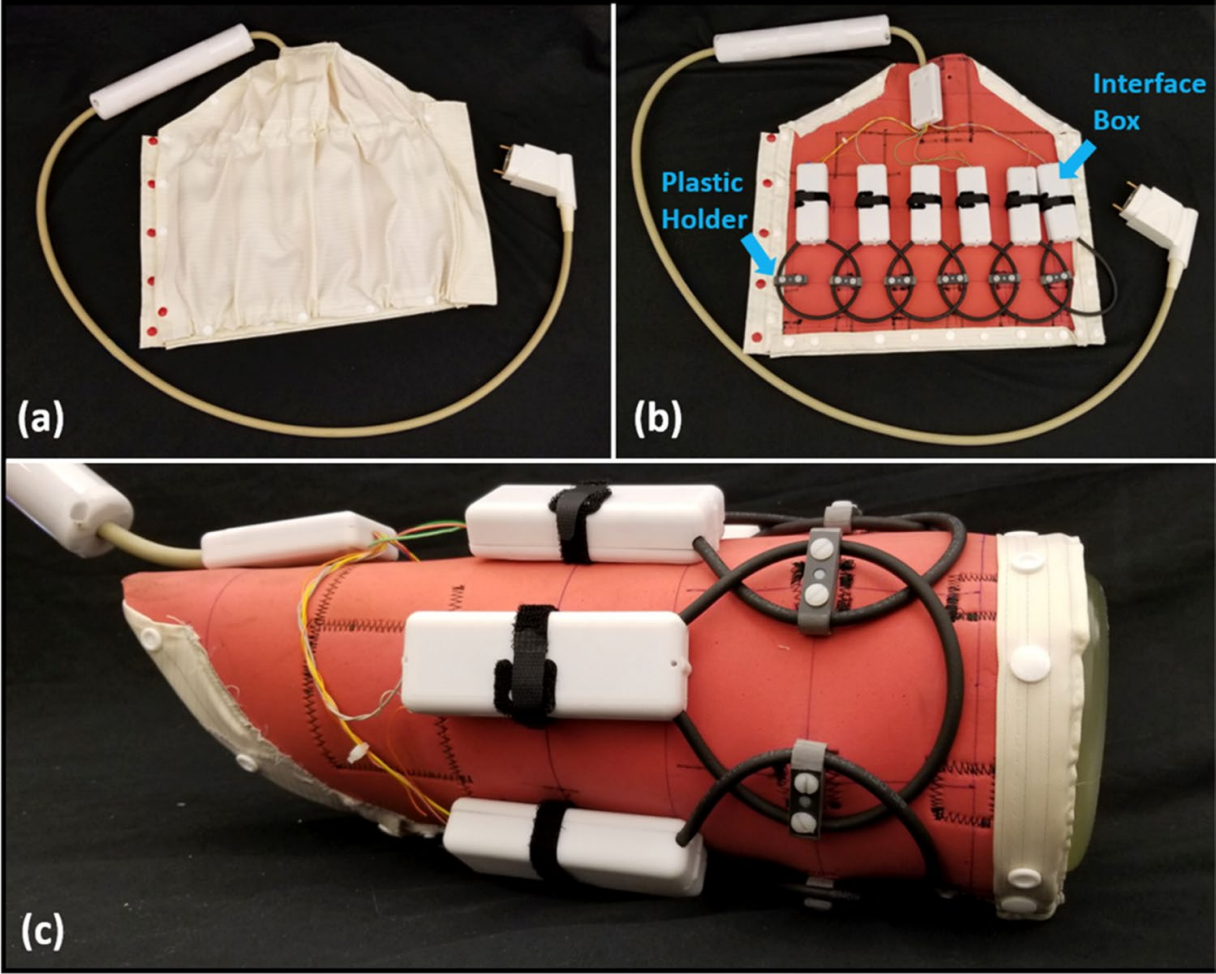
Photograph of the proposed 6-channel flexible coil unrolled with (**a**), without (**b**) the protective cover, and wrapped around a cylindrical phantom (**c**).

**Figure 8:**
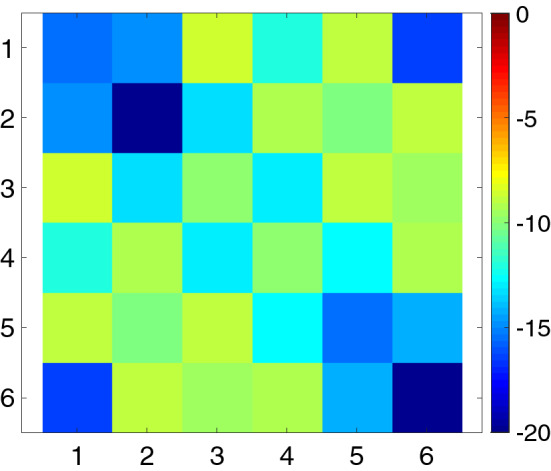
The 6 × 6 scattering matrix of the cable coil wrapped around a phantom as shown in Fig. 7. Scale in dB.

**Figure 9:**
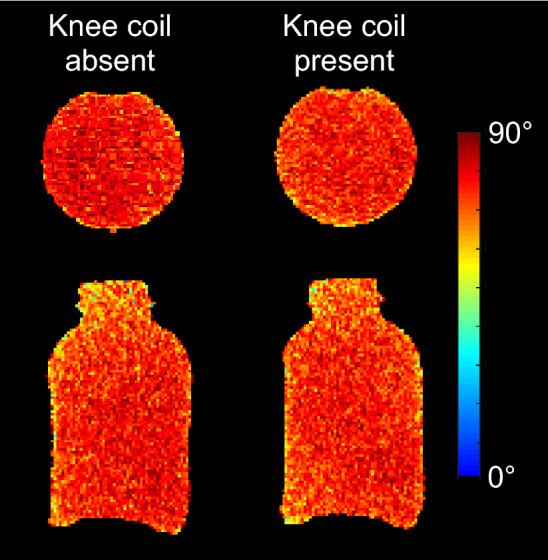
Axial (top row) and sagittal (bottom row) flip angle maps measured with the 6-channel knee coil absent (left) and present (right) illustrate negligible interaction with the system body coil during transmission.

**Figure 10:**
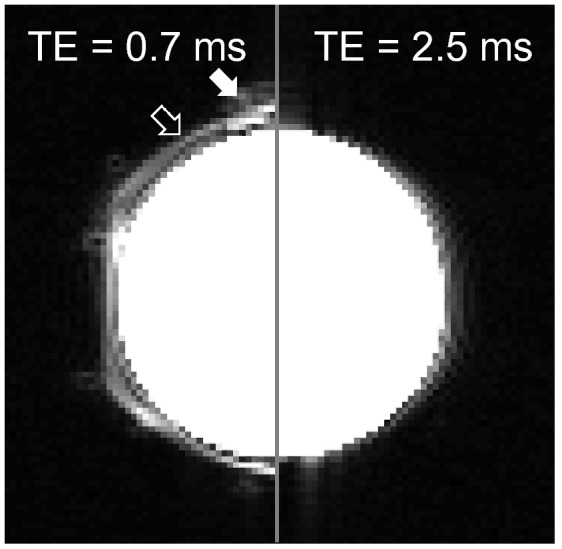
The six-channel cable array included MR-visible components. ABS plastic clamps (solid arrow) and foam cushioning (open arrow) were visible in an image acquired with TE = 0.7 ms (left). The components were not visible with TE = 2.5 ms (right).

Given the suitable bench-top performance and SNR of cable loops, we moved to in vivo scenarios. The cable loop Q values were: Q loaded with the anterior knee = 118 (ratio = 2.6), Q loaded with the posterior knee = 98 (ratio = 3.2). The cable loops were replicated to create a 6-channel array for knee imaging (Fig. 7). The scattering matrix shows that the array was well matched and decoupled (Fig. 8 and Table 2). The Q values of the 6 cable loops in situ (connected to PCBs with matching, detuning, protective fuse and preamplifier circuitry, and in the presence of neighboring coils and cabling) were: unloaded Q = 132 ± 10, Q loaded with a human knee = 95 ± 13, Q ratio = 1.4 ± 0.2. Flip angle maps measured in the presence and absence of the array indicate negligible interaction with the body coil transmitter (Fig. 9). For the same pulse amplitude, the flip angle in a 10-cm diameter ROI in the central axial slice was 77.2 ± 5.9° without the array and 76.6 ± 5.5° with it. The foam cushion and ABS plastic clamps used to house the array were MR visible with TE = 0.7 ms and invisible with TE ≥ 2.5 ms (Fig. 10).

**Table 2:** Summary of the 6 × 6 scattering matrix of the cable coil in Fig. 8.

S-matrix entries	Average (maximum)
Diagonal	− 15.3 (− 9.8) dB
Adjacent neighbors	− 14.0 (− 12.6) dB
Next-adjacent neighbors	− 9.0 (− 8.7) dB
Distant neighbors	− 10.5 (− 9.6) dB

The average (maximum) values are listed for each collection of entries in the matrix.

**Figure 11:**
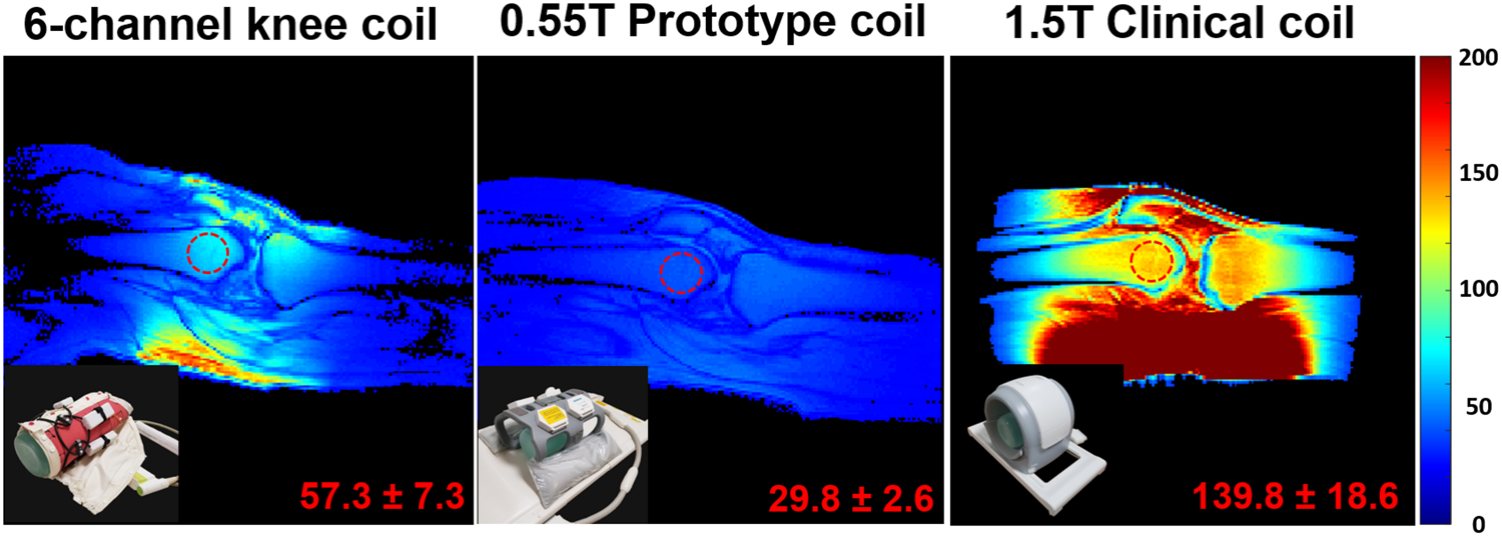
Representative SNR maps acquired with the 0.55 T 6-channel cable coil, 0.55 T prototype coil, and 1.5 T clinical coil. SNR values overlaid in the bottom right are averaged over three volunteers in a 3-cm ROI in the distal femur (overlaid red circles). Coil photos are inset in the bottom left of each panel.

SNR maps show that the 0.55 T cable coil provided approximately twofold gain in the distal femur compared to the makeshift prototype coil that was provided by the scanner manufacturer; 57.3 ± 7.3 versus 29.8 ± 2.6 in three subjects (Fig. 11). To provide another context for comparison, SNR was 139.8 ± 18.6 in the same subjects scanned on a 1.5 T system with a coil used for day-to-day clinical imaging. We scanned another subject three times in the same day (interscan interval of approximately 5 min) to assess repeatability with the 0.55 T cable coil; SNR was 55.2, 55.5, and 58.7 for the three scans (average ± standard deviation was 56.5 ± 1.9).

**Figure 12:**
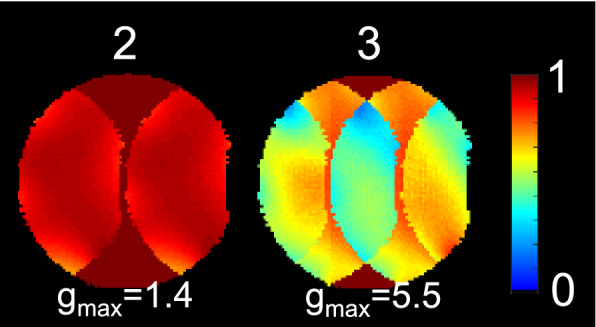
Inverse geometry factor (1/g) maps for twofold and threefold acceleration in the left–right direction in the axial imaging plane. Text overlays indicate the maximum geometry factors (g_max_).

Inverse geometry-factor (1/g) maps^18^ show that the maximum penalty associated with parallel imaging acceleration in the left–right direction was 1.4 for twofold acceleration and 5.5 for threefold acceleration (Fig. 12).

**Figure 13:**
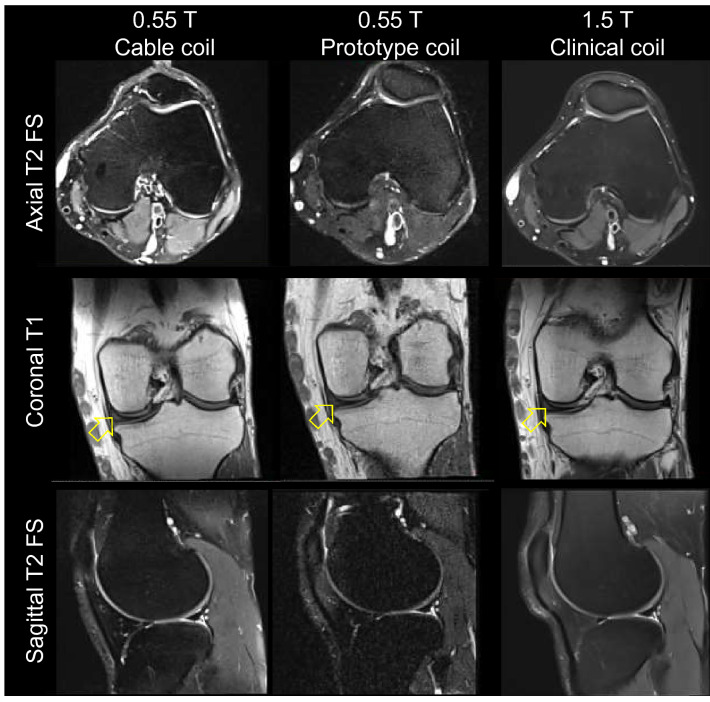
Representative turbo spin echo MR images of a 35-year-old man acquired using the 0.55 T 6-channel cable coil (left column), 0.55 T prototype coil (middle column) and 1.5 T clinical coil (right column). Mucinous degeneration with no meniscal tear is observed in the coronal images (arrows).

**Figure 14:**
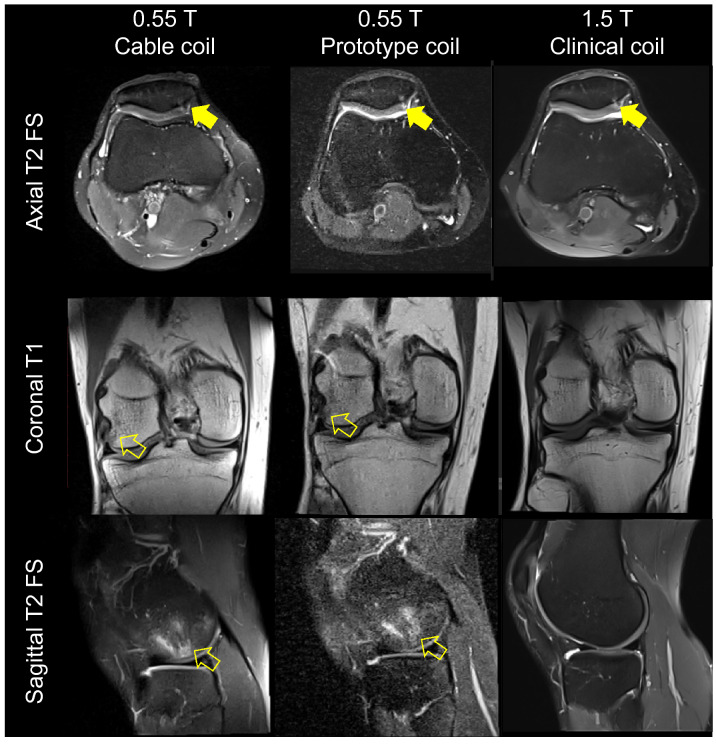
Turbo spin echo MR images of a 34-year-old man acquired using the 0.55 T cable coil (left column), 0.55 T prototype coil (middle column) and 1.5 T clinical coil (right column). The images reveal patellar cartilage defects (solid arrows) and a lateral femoral condyle bone contusion (open arrows). The bone contusion was not present at the time of the 1.5 T examination.

**Figure 15:**
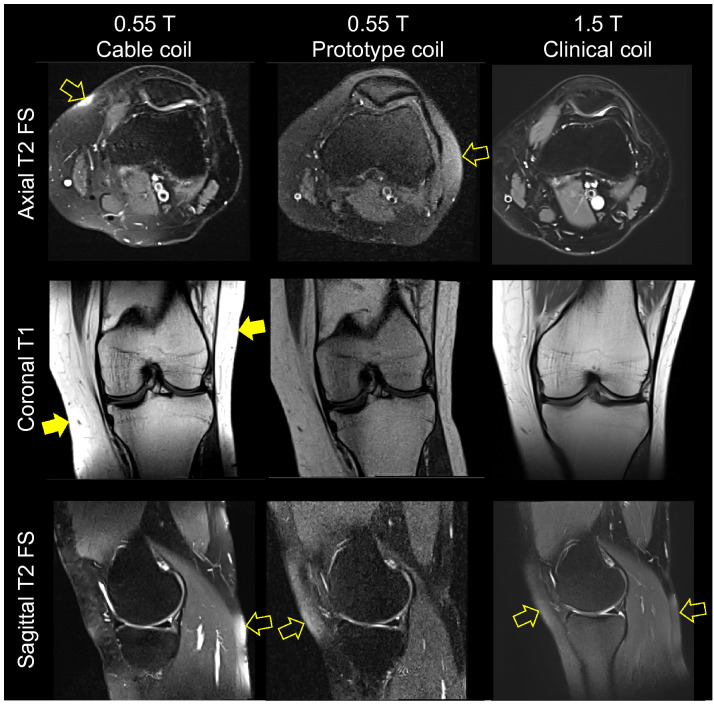
Turbo spin echo MR images from a 28-year-old woman acquired with the 0.55 T 6-channel cable coil (left column), 0.55 T prototype coil (middle column), and 1.5 T clinical coil (right column). The cable coil images have peripheral signal saturation (solid arrows). Images acquired with all coils had heterogeneous fat suppression (hollow arrows).

**Figure 16:**
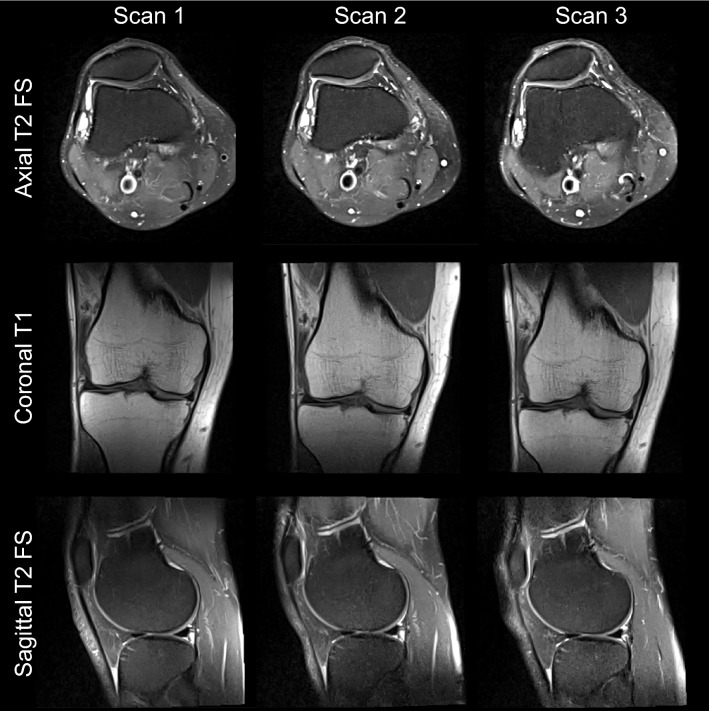
Turbo spin echo MR images from a 31-year-old man acquired with the 0.55 T cable coil during three separate scan sessions on the same day.

Figure 13 shows representative T2-weighted and T1-weighted images of the left knee of a 35-year-old man acquired at 0.55 T with the cable coil and prototype coil and at 1.5 T with the clinical coil. Acceptable image quality was observed in all three cases. The images revealed diffuse intermediate signal within the posterior horn and body of the meniscus consistent with mucinous degeneration with no meniscal tear noted. Figure 14 illustrates cartilage and bone injuries in the right knee of a 34-year-old man. Images of the left knee of a 28-year-old woman show undesirable signal hotspots that are a consequence of the 0.55 T tight-fitting cable coil (Fig. 15). Evidence of heterogeneous fat suppression can also be observed in images acquired with both 0.55 T coils and with the 1.5 T coil to a lesser extent. Image quality was similar in all three sessions of the 0.55 T cable coil repeatability experiment (Fig. 16).

## Discussion

We designed and implemented a knee coil for 0.55 T that utilized flexible conductors formed with the outer shield of RG-223 cables. The cable loops performed similar to semi-rigid Cu-FR4 conductors in terms of SNR. The cable loops also inherited the benefits of coaxial cables such as low cost, widespread availability, ease of assembly, and high durability. While coaxial cables cannot match the stretchability and elasticity of other specialized conductors^3–5,12^, it remains unclear if such features are necessary for routine knee MRI. The selected RG-223 cable has a minimum bend radius of 2.1 cm, which was sufficiently flexible to provide a tight fit to the knee. This flexibility ensured surprisingly good coil-tissue coupling (Q-ratio of 2.6–3.2) considering the relatively low operating frequency of 23.55 MHz and small coil diameter of 10 cm. To put this into context, our group settled for a Q-ratio of only 2 in a previously described semi-flexible six-channel knee coil^19^ despite a higher operating frequency (32.6 MHz vs. 23.55 MHz) and larger loop area (118.5 cm^2^ versus 78.5 cm^2^), highlighting the benefit of the flexible conductor used in the current study. We anticipate that the straightforward concept of forming loops from cable conductors can be translated to other frequencies. Preliminary work from our group showed that a loop formed from the shield of RG-316 cable had a similar Q-ratio as a Cu-FR4 loop at 123.2 MHz (the approximate operating frequency for 3 T MRI systems)^20^.

We found that an individual cable loop provided improved Q-ratio and SNR at 0.55 T over a coaxial loop made from the same RG-223 cable (Table 1, Fig. 2). This trend agrees with 3 T SNR measurements in Fig. 10 in Nohava et al.^21^, in which 15-cm, 10-cm, and 4-cm conventional loops outperformed coaxial loops by 17.1, 28.2, and 48.6%, respectively. Notably, the 3 T conventional loop SNR advantage grew as the loop diameter decreased, suggesting that coaxial conductor loss is greater than that of conventional loops. The additional conductor loss associated with coaxial loops appears to arise from a longer conductor pathway that includes both outer and (smaller) inner conductors. This loss can be significant with respect to sample loss for small loop sizes and/or low operating frequencies. Indeed, Fig. S2 shows that the efficiency of a 10-cm coaxial loop is approximately 25% lower than that of a standard-of-reference Cu-FR4 loop at 23.55 MHz and improves to within 1% at 110 MHz (the coaxial coil self-resonance frequency). Meanwhile, the relative efficiency of the cable loop is the same to within 3% of the Cu-FR4 loop over the same frequency range.

Two-channel coaxial coil arrays provided improved decoupling and tuning robustness against geometrical and overlap variation compared to cable loops (Figs. 3 and 5), which further supports observations made at 3 T by Zhang et al.^6^ and at 7 T by Ruytenberg et al.^14^. However, we found that at 0.55 T, a two-channel cable loop array had 26 or 16% higher SNR than a coaxial array when the elements were overlapped by 20–40% or gapped by 30–50%, respectively (Fig. 6c). One explanation for the apparent discrepancy is that decoupling and tuning have a complex relationship with SNR because of interplay among additional properties such as coil loss, noise correlation, matching circuitry, and preamplifier impedance mismatch^22–30^. From our data on two-channel arrays, we selected cable coils for the six-channel knee array because the plastic hinges (Fig. 7) were expected to maintain approximately 25% overlap between nearest neighbor coils and − 50% overlap between next-nearest neighbors, which correspond to the geometries in which the two-channel cable array outperformed the coaxial array. It is worth noting, however, that these geometric assumptions can breakdown. For example, the two neighbor coils along the seam of the 6-channel array may be incidentally configured with suboptimal overlap for particular knee sizes. Advanced impedance matching techniques could be applied to alleviate SNR degradation in such configurations^3,19,26,29–31^.

We selected RG-223 coaxial cable in this study because it provided low loss and good flexibility. Given that the inner conductor and insulator had little effect on Q of the cable coil, it appears that coaxial cable properties such as characteristic impedance and dielectric constant of the insulator that are important for signal transmission were not relevant for cable coils with 10-cm diameter at 23.55 MHz. This suggests that commercially available flexible single conductor braided wires warrant consideration for forthcoming cable loop designs.

We applied the cable coil concept to knee MRI, which is the most widely accepted imaging tool for diagnosing various knee injuries^32^. Amidst the pursuit of faster scan time and better SNR, many advantages of low-field MRI systems (< 1.0 T) for musculoskeletal radiology, such as reduced metal artifacts^33^ are often overlooked. Recently, a new-generation whole-body 0.55 T MRI system has been cleared by the FDA for clinical imaging^34,35^. The system can offer some of the advantages of dedicated extremity scanners such as greater accessibility due to lower cost compared to high-field systems, improved patient comfort given its 80-cm wide bore, as well as reduced susceptibility artifacts and specific absorption rate^33^.

The complex interplay between magnetic field strength, imaging parameters, tissue relaxation times, acquisition time, SNR, and contrast makes it difficult to arrange a “fair” comparison between images acquired at 0.55 and 1.5 T. Among the various options, we matched as closely as possible the imaging parameters at both fields. While the 1.5 T axial T2 fat suppressed images were visually more appealing than those acquired with the 0.55 T cable coil due to superior tissue contrast and SNR, coronal T1 and sagittal T2 fat suppressed images were visually similar.

Despite being limited by suboptimal receive coil technology, prior studies conducted on low-field whole-body or specialized extremity scanners demonstrated reliable diagnosis of meniscal and ligamentous tears^36–44^. However, SNR challenges may have resulted in difficulties such as inconspicuous cartilage abnormalities depending on field strength and system^39,45^, 5–6 mm thick slices^38,43,44^ that exceeded that set forth in the 2020 American College of Radiology guidelines (4 mm)^46^, or undesirably long examination times^36,43^. In this study, the acquisition time was approximately 3 to 4 min per pulse sequence and the spatial resolution was 0.5 × 0.5 × 3.5 mm. While this study was not intended to evaluate knee joint abnormalities explicitly, cartilage and bone injuries were incidentally delineated, suggesting that clinically acceptable knee examinations are feasible at 0.55 T with a tight-fitting coil that was absent from prior low-field systems.

In reporting that the proposed 6-channel cable coil provided an approximately twofold SNR advantage over the makeshift prototype coil, we acknowledge that the prototype coil is far from optimal for knee imaging. Nonetheless it was the best option provided by the manufacturer, which in fact motivated the current work. To better put the SNR into context, we showed that the 0.55 T cable coil was outperformed by a factor of 2.4 by the 1.5 T 18-channel clinical knee coil, which is less than the threefold difference that is expected from the rule-of-thumb SNR ∝ B_0_^47^. This discrepancy can be explained by T1 differences; T1 of bone is 288 ms at 1.5 T^48^ and estimated to be 203 ms at 0.55 T by applying T1 ∝ B_0_^(0.35)^^49^. Given the gradient echo signal equation^50^ ${\text{S = }}\frac{{{\text{sin}}\;{\text{FA}}\left( {{\text{1 - e}}^{{\text{ - TR/T1}}} } \right)}}{{{\text{1 - cos}}\;{\text{FA}} \cdot {\text{e}}^{{\text{ - TR/T1}}} }}$, where FA is flip angle, the T1 difference is expected to translate into a 25% SNR advantage at 0.55 T for the imaging parameters selected in this study (TR = 200 ms and flip angle = 20°). After correcting for T1, we find a 3.0-fold SNR advantage at 1.5 T, which suggests that the 0.55 T cable coil performed as expected and conductor loss does not deleteriously impact SNR, which might be anticipated at low field.

One drawback of the cable coil is that its tight fit resulted in signal hotspots, particularly in T1w images. Saturated signal was evident in the knee periphery (Fig. 15) despite a 5 mm foam spacer in the coil housing that was intended to improve uniformity. Further study is required to determine whether clinical readability is impacted or whether image uniformity can be improved by methods such as bias field correction in post-processing^51^.

The foam spacer and ABS plastic clamps were MR-visible for TE < 2.5 ms. While this can be undesirable for ultra-short TE applications such as bound water imaging, it is not anticipated to be problematic for conventional or rapid knee imaging protocols that primarily utilize spin echo sequences with TEs on the order of 10 ms or longer^52^.

We observed non-uniform fat suppression in 0.55 T T2-weighted images; at the time of writing, it is unclear whether this is a subject-specific or a systematic issue since poor fat suppression was observed in images acquired with the cable and prototype coils. Given the narrow spectral separation between fat and water at 0.55 T (80 Hz), the simplistic fat suppression technique used here may be outperformed by methods that utilize adiabatic pulses and inversion recovery modules, for example, SPAIR (spectrally selective adiabatic inversion recovery).

The chosen coil dimensions (6 loops of 10 cm diameter that were overlapped for decoupling) were suitable for encircling a cylinder with 13.5 cm diameter, which is similar to the 12.8-cm diameter of the 99th percentile male knee^53^. However, in practice, the distal thigh is the limiting factor for accessibility. While we found that our design accommodated the four enrolled volunteers with BMI = 23.9, 25.2, 25.2, and 30.7 kg/m^2^ (Figs. 13, 14, 15, 16), we acknowledge that the coil may not fully encircle larger subjects, which could compromise image quality. To accommodate a greater portion of the population, a future design may utilize slightly larger loops or foam cutouts that are fitted to the loops rather than the continuous foam sheet in the current coil.

The coil presented here has a single-row layout that makes it sensitive to placement and incapable of longitudinal parallel imaging acceleration^52^. In principle, these limitations could be overcome by replicating the existing single-row layout to form an "Olympic ring" two-row array similar to that in Rispoli et al.^54^. Such a layout may deserve future exploration.

In conclusion, the proposed cable coil utilized flexible, commercially available conductors, which showed comparable SNR as a reference Cu-FR4 loop and adequate SNR robustness against geometric variability. While cable loops do not provide elastic properties that have been demonstrated elsewhere^4,5^, cables are relatively inexpensive (on the order of $20 per meter) and allow straightforward coil assembly. Applied to knee imaging, the cable coil array provided promising image quality, particularly in the cartilage, which has been difficult to examine with older generation low-field systems^39,45^, in a clinically acceptable examination time^36,43^. Natural extensions of this work will be to apply the coaxial shield conductor concept to other anatomies and other operating frequencies and to rigorously evaluate image quality and diagnostic accuracy in a larger cohort.

## Methods

The loops were made to resonate at 23.55 MHz by inserting one capacitor in series with RG-223 (part number 9273, Belden, St. Louis, MO) or 1 oz. copper plated FR4 circuit board (Cu-FR4) with 11-mm trace width (Fig. 1). All loops were 10-cm in diameter. For the cable loop, the capacitor was in series with the outer shield, while the inner conductor was broken at the feed port but not connected (electrically floating). For the coaxial loop, gaps were placed in the RG-223 shield and inner conductor at opposite locations along the circumference, and tuning was carried out using a capacitor in the inner conductor gap. The Q-values were measured using a vector network analyzer (model E5070B, Agilent, Santa Clara, CA) with the loops in free space and while loaded with a cylindrical phantom (diameter = 13.5 cm, volume = 3.4 L) that contained 2.8 g NiSO_4_ and 2.7 g NaCl per 1 L water or with a human knee.

Scattering matrix and imaging measurements were performed with each coil connected to a circuit board that contained a preamplifier port and components for matching, detuning, and preamplifier decoupling. Preamplifier decoupling efficacy was 20 to 25 dB for the cable loops and coaxial loops. This measurement was defined as the difference between S_21_ measured with a double pickup probe coupled lightly to the coil without and with the preamplifier present. The cable and coaxial loop detuning circuits provided at least 30 dB isolation. This measurement was defined as the difference between S_21_ measured with a double pickup probe coupled lightly to the coil with the detuning circuit inactive and active.

Sensitivity to geometric variability was investigated by measuring the reflection coefficient at the preamplifier port on a cable loop and a coaxial loop that were tuned with 10-cm diameter circular contours. Without adjusting the tuning, the measurement was repeated after elongating the loops into ellipses whose major axes were 13-cm and 15-cm. Sensitivity to overlap was investigated by measuring reflection and transmission coefficients at the preamplifier ports on two-channel arrays based on cable or coaxial loops. The coils were tuned in isolation. Without adjusting the tuning, the reflection and transmission coefficients were measured when the two-channel array elements were overlapped from − 50 to + 50% in 5% steps.

MRI data were acquired on two commercial systems: (1) a 1.5 T MAGNETOM Aera (Siemens Healthcare, Erlangen, Germany) that was modified to operate as a prototype at 0.55 T as described by Campbell-Washburn et al.^55^ or (2) a 1.5 T MAGNETOM Sola that operated as usual. SNR maps were measured in the phantom with one or two-channel arrays based on 10-cm cable, coaxial, and Cu-FR4 loops. For the two-channel arrays, measurements were performed with overlap between the loops ranging from + 50 to − 50% in 10% steps. SNR maps were calculated from signal and noise (with the RF pulse amplitude set to zero) measurements acquired with a gradient echo pulse sequence and processed with the optimal array combination method^1,56^. The imaging parameters for SNR measurements were: repetition time (TR) = 200 ms, echo time (TE) = 5.8 ms, flip angle = 20°, voxel size = 1.6 × 1.6 × 10 mm^3^, pixel bandwidth (BW) = 130 Hz/pixel, and acquisition time (TA) = 38 s. The phantom SNR maps were reconstructed with 2.3 × 2.3 × 10 mm^3^ voxels. Geometry factor maps^18^ were calculated from the same data using methods described by Montin and Lattanzi^57^.

Six cable loops were arranged to form a knee coil array. Fuses were added to the circuit boards to prevent current flow during body coil transmission in the event of an active detuning circuit failure. The boards were enclosed in rigid plastic and the assembly in flame-resistant fabric (Fig. 7). Adjacent loops were linked together by ABS plastic polymer (acrylonitrile butadiene styrene) hinges that allowed mechanical flexibility while maintaining approximate geometric overlap for inductive decoupling. A quarter-wave cable trap (“bazooka” balun, 20-cm length, and 3-cm diameter)^58^ was installed on the bundled cable between the coil elements and scanner interface to reduce common mode currents.

Flip angle maps were acquired using a 2D turbo flash (fast low angle shot) sequence with a preparation pulse and the following parameters: transmit reference voltage = 270 v, TR = 10,000 ms, TE = 1.8 ms, imaging flip angle = 15°, saturation flip angle = 80°, voxel size = 2.3 × 2.3 × 20 mm^3^, BW = 490 Hz/pixel, and TA = 20 s. A 3D turbo flash sequence with the following parameters was used to reveal MR-visible components: TR = 4.2 ms, TE = 0.7 and 2.5 ms, flip angle = 25°, voxel size = 2.3 × 2.3 × 5 mm^3^, BW = 1560 Hz/pixel, and TA = 1:09 min.

Knee MRI was performed in 3 configurations: (1) at 0.55 T with the proposed six-channel array; (2) at 0.55 T with a 6-channel body coil in combination with an 18-channel spine coil that were originally built for 1.5 T and subsequently re-tuned by the manufacturer to 23.55 MHz (referred to as the “0.55 T prototype coil”); and (3) at 1.5 T with a state-of-the-art product coil used for clinical imaging (“TxRx Knee 18”, Siemens Healthineers, Erlangen, Germany). For case 2, the coils were repurposed for knee imaging by wrapping the body coil around the anterior knee and using the spine coil to cover the posterior knee. The body coil consists of 6 loops (approximately 16 cm × 18 cm) arranged into 2 rows of 3, while the spine coil consists of 18 loops (approximately 16 cm × 16 cm) arranged into 6 rows of 3. Note that the body coil was not sufficiently flexible to tightly fit the knee; its minimum bend radius was approximately 11 cm.

The study was fully compliant with the Health Insurance Portability and Accountability Act and the New York University Institutional Review Board approved the protocol. All experiments were performed in accordance with relevant guidelines and regulations. We scanned four human subjects after obtaining their informed written consent. We performed 2D turbo spin echo imaging to evaluate the efficacy of the coils for clinical research. The 0.55 T knee imaging parameters were chosen to meet American College of Radiology specifications^46^ and are as follows: axial T2-weighted with fat suppression with TR = 3300 ms, TE = 35 ms, echo train length (ETL) = 11, voxel size = 0.5 × 0.5 × 3.5 mm^3^, BW = 130 Hz/pixel, parallel imaging acceleration factor (iPAT) = 2, number of signal averages = 2, TA = 4:03 min; coronal T1-weighted with TR = 533 ms, TE = 12 ms, ETL = 2, voxel size = 0.5 × 0.5 × 3.5 mm^3^, BW = 150 Hz/pixel, iPAT = 2, number of signal averages = 2, TA = 3:18 min and; sagittal T2-weighted with fat suppression with TR = 3400 ms, TE = 35 ms, ETL = 11, voxel size = 0.5 × 0.5 × 3.5 mm^3^, BW = 130 Hz/pixel, iPAT = 2, number of signal averages = 2, TA = 4:00 min. Fat suppression was performed with the product spectrally selective saturation method. The 1.5 T imaging parameters were matched as closely as possible to those at 0.55 T: axial T2-weighted with fat suppression with TR = 3300 ms, TE = 36 ms, echo train length (ETL) = 11, voxel size = 0.5 × 0.5 × 3.5 mm^3^, BW = 129 Hz/pixel, parallel imaging acceleration factor (iPAT) = 2, number of signal averages = 2, TA = 3:51 min; coronal T1-weighted with TR = 545 ms, TE = 11 ms, ETL = 2, voxel size = 0.5 × 0.5 × 3.5 mm^3^, BW = 150 Hz/pixel, iPAT = 2, number of signal averages = 2, TA = 3:19 min and; sagittal T2-weighted with fat suppression with TR = 3400 ms, TE = 34 ms, ETL = 11, voxel size = 0.5 × 0.5 × 3.5 mm^3^, BW = 130 Hz/pixel, iPAT = 2, number of signal averages = 2, TA = 3:53 min.

## Supplementary Information

Supplementary Information.

## Data Availability

The datasets generated during and/or analyzed during the current study are available from the corresponding author on reasonable request.

## References

[1] Roemer PB, Edelstein WA, Hayes CE, Souza SP, Mueller OM (1990). The NMR phased array. Magn. Reson. Med..

[2] Zhang B (2019). Size-adaptable "Trellis" structure for tailored MRI coil arrays. Magn. Reson. Med..

[3] Nordmeyer-Massner JA, De Zanche N, Pruessmann KP (2012). Stretchable coil arrays: Application to knee imaging under varying flexion angles. Magn. Reson. Med..

[4] Port A (2020). Detector clothes for MRI: A wearable array receiver based on liquid metal in elastic tubes. Sci. Rep..

[5] Port A, Luechinger R, Brunner DO, Pruessmann KP (2021). Elastomer coils for wearable MR detection. Magn. Reson. Med..

[6] Zhang B, Sodickson DK, Cloos MA (2018). A high-impedance detector-array glove for magnetic resonance imaging of the hand. Nat. Biomed. Eng..

[7] Zhang B (2022). Twenty-four-channel high-impedance glove array for hand and wrist MRI at 3T. Magn. Reson. Med..

[8] Darnell D, Truong TK, Song AW (2022). Recent advances in radio-frequency coil technologies: Flexible, wireless, and integrated coil arrays. J. Magn. Reson. Imaging.

[9] Corea JR (2016). Screen-printed flexible MRI receive coils. Nat. Commun..

[10] Corea JR, Lechene PB, Lustig M, Arias AC (2017). Materials and methods for higher performance screen-printed flexible MRI receive coils. Magn. Reson. Med..

[11] Jia F (2015). Knee MRI under varying flexion angles utilizing a flexible flat cable antenna. NMR Biomed..

[12] Vincent JM, Rispoli JV (2019). Conductive thread-based stretchableand flexible radiofrequency coils for magnetic resonance imaging. IEEE Trans. Biomed. Eng..

[13] Harpen MD (1994). The theory of shielded loop resonators. Magn. Reson. Med..

[14] Stensgaard A (1996). Optimized design of the shielded-loop resonator. J. Magn. Reson. A.

[15] Ruytenberg T, Webb A, Zivkovic I (2020). Shielded-coaxial-cable coils as receive and transceive array elements for 7T human MRI. Magn. Reson. Med..

[16] Axel L, Hayes C (1985). Surface coil magnetic resonance imaging. Arch. Int. Physiol. Biochim.

[17] Hayes CE, Axel L (1985). Noise performance of surface coils for magnetic resonance imaging at 1.5 T. Med. Phys..

[18] Pruessmann KP, Weiger M, Scheidegger MB, Boesiger P (1999). SENSE: sensitivity encoding for fast MRI. Magn. Reson. Med..

[19] Brown R (2016). A flexible nested sodium and proton coil array with wideband matching for knee cartilage MRI at 3T. Magn. Reson. Med..

[20] Siddiq, S. S., Lakshmanan, K., Walczyk, J., Bruno, M. & Brown, R. Wearable coil for knee flexion MRI. In *Proceedings of the IEEE International Conference on Electromagnetics in Advanced Applications* 378 (2021).

[21] Nohava L (2021). Flexible multi-turn multi-gap coaxial RF coils: Design concept and implementation for magnetic resonance imaging at 3 and 7 Tesla. IEEE Trans. Med. Imaging.

[22] Reykowski A, Wright SM, Porter JR (1995). Design of matching networks for low noise preamplifiers. Magn. Reson. Med..

[23] Nordmeyer-Massner JA, De Zanche N, Pruessmann KP (2009). Mechanically adjustable coil array for wrist MRI. Magn. Reson. Med..

[24] Brown R, Wang Y, Spincemaille P, Lee RF (2007). On the noise correlation matrix for multiple radio frequency coils. Magn. Res. Med..

[25] Stumpf C, Malzacher M, Schmidt L-P (2018). Radio frequency modeling of receive coil arrays for magnetic resonance imaging. J. Imaging.

[26] Vester, M. *et al.* Mitigation of inductive coupling in array coils by wideband port matching. In *Proceedings of ISMRM* 2690 (2012).

[27] Reykowski, A., Saylor, C. & Duensing, G. R. Do we need preamplifier decoupling. In *Proceedings of ISMRM* 3883 (2011).

[28] Reykowski, A. & Wang, J. Rigid SNR analysis of coupled MRI coils connected to noisy preamplifiers and the effect of coil decoupling on combined SNR. In *Proceedings of ISMRM* 1402 (2000).

[29] Findeklee C (2011). Array noise matching—generalization, proof and analogy to power matching. IEEE Trans. Antennas Propagat..

[30] Sanchez-Heredia JD (2018). Improved decoupling for low frequency MRI arrays using non-conventional preamplifier impedance. IEEE Trans. Biomed. Eng..

[31] Wiggins, G. C. *et al.* SNR degradation in receive arrays due to preamplifier noise coupling and a method for mitigation. In *Proceedings of ISMRM* 2689 (2012).

[32] Khodarahmi I, Fritz J (2021). The value of 3 Tesla field strength for musculoskeletal magnetic resonance imaging. Invest. Radiol..

[33] Khodarahmi I (2022). New-generation low-field magnetic resonance imaging of hip arthroplasty implants using slice encoding for metal artifact correction: First in vitro experience at 0.55 T and comparison with 1.5 T. Invest. Radiol..

[34] Siemens Healthineers Announces FDA Clearance of MAGNETOM Free.Max 80 cm MR Scanner. https://www.siemens-healthineers.com/en-us/press-room/press-releases/fdaclearsmagnetomfreemax.html (2020).

[35] 21 CFR Part 892. Magnetic resonance diagnostic device. <https://www.accessdata.fda.gov/cdrh_docs/pdf21/K210611.pdf (2021).

[36] Lee CS, Davis SM, McGroder C, Stetson WB, Powell SE (2013). Analysis of low-field magnetic resonance imaging scanners for evaluation of knee pathology based on arthroscopy. Orthop. J. Sports Med..

[37] Kladny B, Gluckert K, Swoboda B, Beyer W, Weseloh G (1995). Comparison of low-field (02 Tesla) and high-field (15 Tesla) magnetic resonance imaging of the knee joint. Arch. Orthop. Trauma Surg..

[38] Riel KA, Reinisch M, Kersting-Sommerhoff B, Hof N, Merl T (1999). 0.2-Tesla magnetic resonance imaging of internal lesions of the knee joint: A prospective arthroscopically controlled clinical study. Knee Surg. Sports Traumatol. Arthrosc..

[39] Leigheb M (2018). Role of low field MRI in detecting knee lesions. Acta Biomed..

[40] Barnett MJ (1993). MR diagnosis of internal derangements of the knee: Effect of field strength on efficacy. AJR Am. J. Roentgenol..

[41] Cotten A (2000). MR imaging of the knee at 0.2 and 1.5 T: Correlation with surgery. AJR Am. J. Roentgenol..

[42] Franklin PD, Lemon RA, Barden HS (1997). Accuracy of imaging the menisci on an in-office, dedicated, magnetic resonance imaging extremity system. Am. J. Sports Med..

[43] Kinnunen J (1994). Diagnostic performance of low field MRI in acute knee injuries. Magn. Reson. Imaging.

[44] Vellet AD (1995). Anterior cruciate ligament tear: Prospective evaluation of diagnostic accuracy of middle- and high-field-strength MR imaging at 1.5 and 0.5 T. Radiology.

[45] Ghazinoor S, Crues JV, Crowley C (2007). Low-field musculoskeletal MRI. J. Magn. Reson. Imaging.

[46] ACR–SPR–SSR practice parameter for the performance and interpretation of magnetic resonance imaging of the knee. https://www.acr.org/-/media/ACR/Files/Practice-Parameters/mr-knee.pdf (2020).

[47] Haacke EM, Brown RW, Thompson MR, Venkatesan R (1999). Magnetic Resonance Imaging—Physical Principles and Sequence Design.

[48] Gold GE (2004). Musculoskeletal MRI at 3.0 T: Relaxation times and image contrast. AJR Am. J. Roentgenol..

[49] Rooney WD (2007). Magnetic field and tissue dependencies of human brain longitudinal 1H2O relaxation in vivo. Magn. Reson. Med..

[50] Liang, Z. P., Lauterbur, P. C. & IEEE Engineering in Medicine and Biology Society. *Principles of Magnetic Resonance Imaging: A Signal Processing Perspective* (SPIE Optical Engineering Press, IEEE Press, 2000).

[51] Tustison NJ (2010). N4ITK: Improved N3 bias correction. IEEE Trans. Med. Imaging.

[52] Fritz J, Guggenberger R, Del Grande F (2021). Rapid musculoskeletal MRI in 2021: Clinical application of advanced accelerated techniques. AJR Am. J. Roentgenol..

[53] Tilley AR (1993). The Measure of Man and Woman.

[54] Rispoli JV, Wilcox MD, By S, Wright SM, McDougall MP (2016). Effects of coplanar shielding for high field MRI. Annu. Int. Conf. IEEE Eng. Med. Biol. Soc..

[55] Campbell-Washburn AE (2019). Opportunities in interventional and diagnostic imaging by using high-performance low-field-strength MRI. Radiology.

[56] Kellman P, McVeigh ER (2005). Image reconstruction in SNR units: A general method for SNR measurement. Magn. Reson. Med..

[57] Montin E, Lattanzi R (2021). Seeking a widely adoptable practical standard to estimate signal-to-noise ratio in magnetic resonance imaging for multiple-coil reconstructions. J. Magn. Reson. Imaging.

[58] Schaller, B. M., Magill, A. W. & Gruetter, R. Common modes and cable traps. In *Proceedings of the International Society for Magnetic Resonance in Medicine* 4660 (2011).

