# Supplementary material for: A flexible MRI coil based on a cable conductor and applied to knee imaging
Source: Sci Rep. 2022 Sep 2;12:15010. doi: 10.1038/s41598-022-19282-6 (PMC9440226; doi:10.1038/s41598-022-19282-6)
Supplement: Supplementary file 1 — Supplementary Information. [file 41598_2022_19282_MOESM1_ESM.pdf]

A flexible MRI coil based on a cable conductor and applied to knee imaging

We tuned 10-cm Cu-FR4, cable, and coaxial loops to a range of frequencies (23.55 to 110 MHz) by adjusting the capacitors in Figure S1. At each frequency, we measured the loaded and unloaded Q values. The load was a cylindrical phantom (diameter = 13.5 cm, volume = 3.4 L) that contained 2.8 g $NiSO_4$ and 2.7 g NaCl per 1 L water. The unloaded-to-loaded Q ratio was used to calculate the relative efficiency as defined by $\sqrt{1 - \text{Q ratio}^{-1}}$ [1-3]. Figure S2 shows that the efficiency of a 10-cm coaxial loop is approximately 25% lower than that of a standard-of-reference Cu-FR4 loop at 23.55 MHz and improves to within 1% at 110 MHz (the coaxial coil self-resonance frequency). Meanwhile, the relative efficiency of the cable loop is the same to within 3% of the Cu-FR4 loop over the same frequency range.

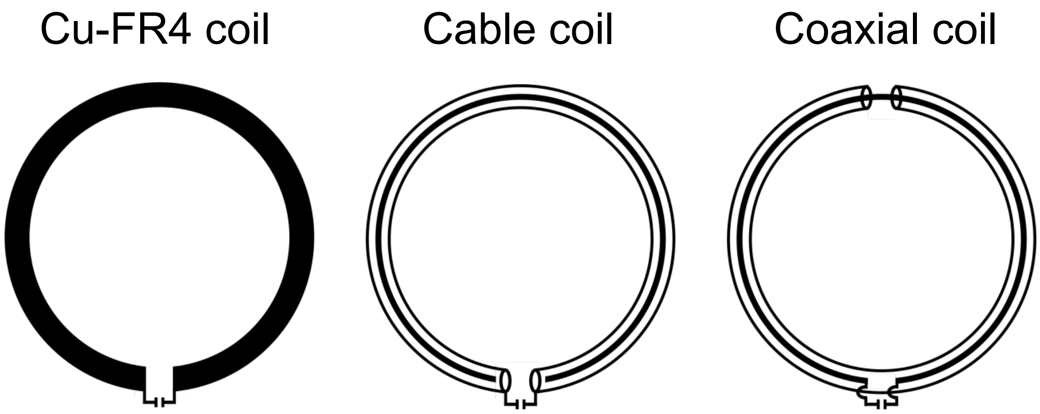

Figure S1**.** Coil schematics for Q measurements.

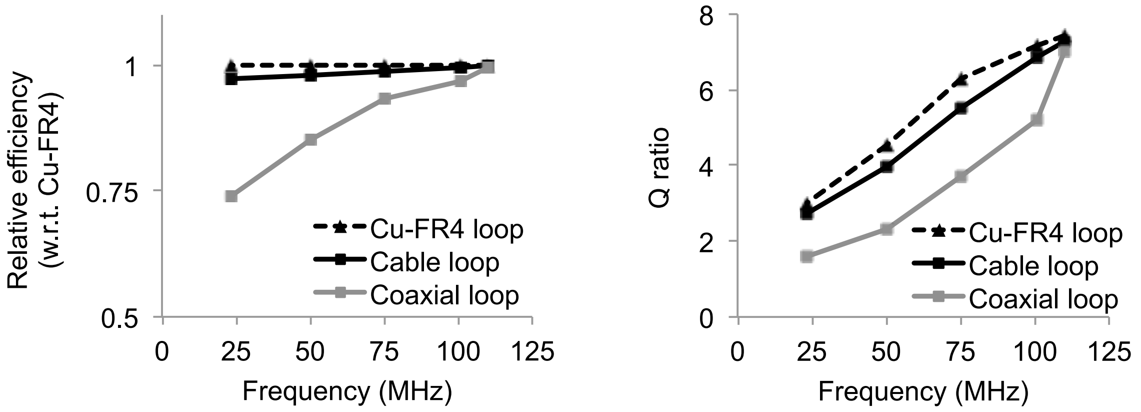

Figure S2. Relative efficiency (left) and Q ratio (right) as a function of frequency for 10-cm Cu-FR4, cable, and coaxial loops.

REFERENCES

1 Port, A., Luechinger, R., Brunner, D. O. & Pruessmann, K. P. Elastomer coils for wearable MR detection. *Magn Reson Med* **85**, 2882-2891, doi:10.1002/mrm.28662 (2021).

2 Axel, L. & Hayes, C. Surface coil magnetic resonance imaging. *Arch Int Physiol Biochim* **93**, 11-18 (1985).

3 Hayes, C. E. & Axel, L. Noise performance of surface coils for magnetic resonance imaging at 1.5 T. *Med Phys* **12**, 604-607 (1985).
